# In the *IMD2* gene of *Saccharomyces cerevisiae*, the expression memory suppresses the induction of expression during guanosine triphosphate depletion

**DOI:** 10.1093/pnasnexus/pgag179

**Published:** 2026-05-22

**Authors:** Takuma Yokosawa, Takahito Ayano, Masaya Oki

**Affiliations:** Department of Applied Chemistry and Biotechnology, Graduate School of Engineering, University of Fukui, Fukui 910-8507, Japan; Department of Applied Chemistry and Biotechnology, Graduate School of Engineering, University of Fukui, Fukui 910-8507, Japan; Research Fellowships of Japan Society for the Promotion of Science for Young Scientists (JSPS), Tokyo 102-0083, Japan; Department of Applied Chemistry and Biotechnology, Graduate School of Engineering, University of Fukui, Fukui 910-8507, Japan; Life Science Innovation Center, University of Fukui, Fukui 910-8507, Japan

**Keywords:** epigenetics, chromatin, memory, GTP, single cell

## Abstract

In budding yeast, genes located within the heterochromatin regions are transcriptionally repressed. The boundaries of heterochromatin regions fluctuate in response to intracellular and extracellular influences, thereby altering the gene expression in nearby regions. However, the mechanisms regulating chromatin organization at specific loci are unclear. Therefore, we used a single-cell tracking system to analyze *IMD2*, a gene located in the subtelomeric regions, whose expression is regulated by variations in heterochromatin structure. While the heterochromatin region at the *IMD2* locus fluctuates to induce expression in response to guanosine triphosphate (GTP) depletion, we found that *IMD2* expression states change independently of GTP levels in a subset of cells. When the *IMD2* expression state was tracked before and after GTP depletion, cells that had previously exhibited *IMD2* expression were less likely to show fluctuations in heterochromatin state following GTP depletion. These results suggest that the heterogeneous regulation of *IMD2* expression observed at the cell population level is influenced by prior transcriptional experience, which is associated with variations in the heterochromatin structure.

Significance statement One definition of “cellular memory” refers to the persistence of chromatin structural changes in the epigenetic regulation of gene expression. It is generally believed that once the chromatin structure opens and gene expression is turned ON in a heterochromatic region, where transcription is typically repressed, chromatin becomes easier to open. However, this study identifies a phenomenon that deviates from this conventional model: once gene expression is induced in a heterochromatic region, subsequent re-induction is suppressed as the region becomes more resistant to reactivation.

## Introduction

Eukaryotic gene expression is regulated by chromatin structure. In general, genes are expressed in euchromatic regions, where chromatin is open, whereas gene expression is repressed in heterochromatic regions, where chromatin is closed (1). Chromatin structure is stable and faithfully maintained through mitotic cell divisions (2). In budding yeast, the *HM*, telomere, and rDNA regions are known sites of heterochromatin formation (3–5). In the *HM* and telomere regions, Sir2 deacetylates H4K16, leading to the spreading of Sir2, Sir3, and Sir4 complexes along chromosomes, thereby forming and maintaining heterochromatin (3, 6). Continued spreading of the heterochromatin region by the Sir protein complex represses the expression of essential genes; therefore, a boundary is formed to limit spreading (7–11). Single-cell tracking experiments in budding yeast revealed that heterochromatin boundaries are regulated by boundary-forming factors, leading to changes in the expression of adjacent genes (12). In this study, we focused on *IMD2*, a gene located near the telomere on the right arm of chromosome VIII, whose expression is regulated by fluctuations in the heterochromatin boundaries (13). *IMD2* encodes inosinate dehydrogenase (IMPDH), which catalyzes the conversion of inosinic acid (IMP) to xanthosinic acid (XMP) in the de novo guanosine triphosphate (GTP) biosynthesis pathway (14). Four genes encode inosinate dehydrogenases (*IMD1*, *IMD2*, *IMD3*, and *IMD4*), with *IMD3* and *IMD4* being constitutively expressed, whereas *IMD1* and *IMD2* are repressed. However, *IMD1* is known to be nonfunctional because of a frameshift in its open-reading frame (ORF) (15, 16). *IMD2* confers resistance to mycophenolic acid (MPA), an inhibitor of IMPDH, and is essential for growth under high MPA (17–19). *IMD2* expression was also regulated in a DNA sequence–dependent manner in response to GTP depletion. When GTP is abundant in vivo, transcription is initiated upstream of the repressor element (RE) region, and the resulting transcripts are attenuated, leading to the repression of *IMD2* expression (20–22). In contrast, under GTP-depleted conditions, the transcription start site shifts from upstream to downstream of the RE region, resulting in *IMD2* expression (23–28). Thus, the expression of *IMD2* is regulated to maintain intracellular GTP levels at a constant value. However, *IMD2* is subject to differential epigenetic regulation between cells and is variably expressed in some cells even when GTP levels are maintained at a constant level under identical culture conditions (13). Heterogeneity in expression was also observed during GTP depletion, suggesting the presence of a system that generates a minority of cells with distinct expression states within the cell population.

The mechanism by which a small number of cells in a population adopt different states, even under uniform environmental conditions, has been reported in budding yeast, as well as in higher eukaryotes. For example, in mouse embryonic stem cells, the expression levels of *Zscan4* fluctuate heterogeneously among individual cells, and it has been reported that the transient expression of *Zscan4* in each cell contributes to the maintenance of genetic stability at the population level (29–31). However, in yeast, the physiological significance of the fluctuating heterochromatin regions in response to GTP levels in vivo and the maintenance of a minority of such cells within the population remain unclear.

In this study, we employed a stepwise approach using single-cell analysis with fluorescent protein reporters to elucidate the mechanisms by which boundary fluctuations of heterochromatin in subtelomeric regions result in a heterogeneous gene expression. First, to clarify how the initial expression state (ON or OFF) of *IMD2* influences subsequent expression regulation, we defined these states at the population level. Furthermore, we investigated how continuous fluctuations in the expression levels of individual cells affect the subsequent transcriptional control. Through these analyses, we demonstrated that dynamic fluctuations at the boundaries of heterochromatin regions are one of the key factors generating cell-to-cell heterogeneity in gene expression states.

## Results

### The expression distribution of *IMD2* in cell populations changed depending on the concentration of MPA, which induced GTP depletion

**Figure 1:**
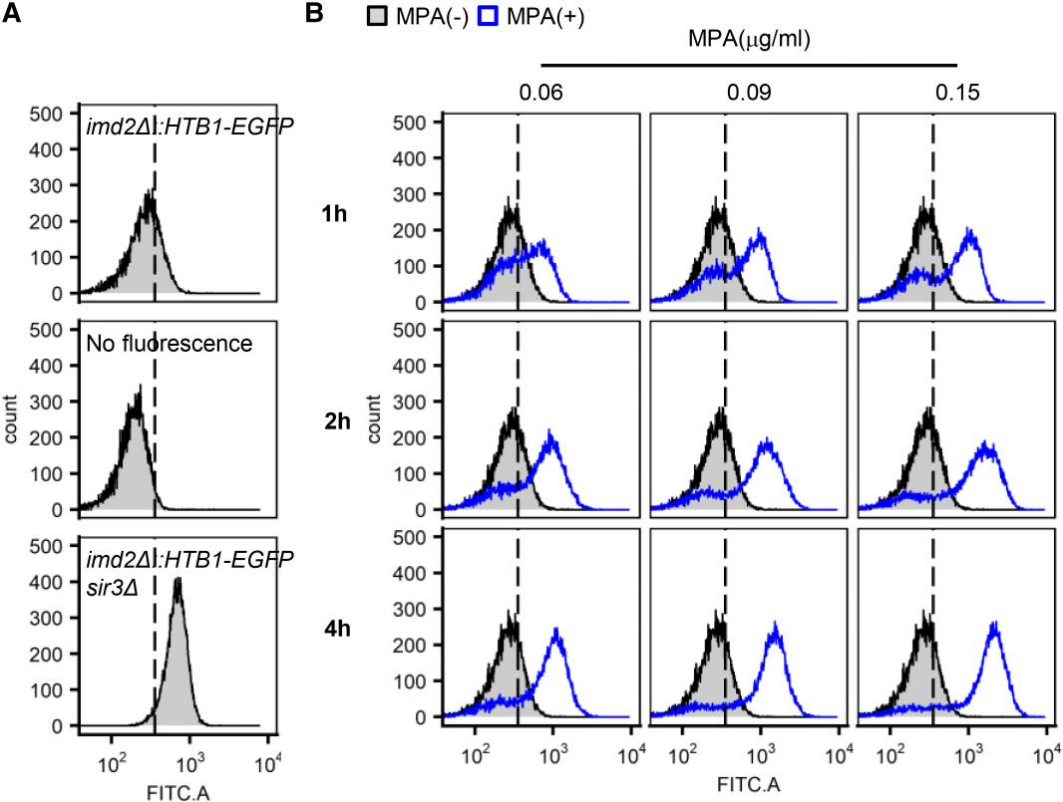
The expression distribution of *IMD2* in cell populations changed depending on the concentration of MPA, which induces GTP depletion. The distribution of *IMD2* expression in cell populations was analyzed by measuring the EGFP fluorescence intensity of yeast strains, which changes in a GTP-dependent manner, using FCM. Each sample was analyzed using 20,000 cells and plotted as a histogram. The horizontal axis indicates the fluorescence intensity, and the vertical axis represents the total cell count. The thresholds for OFF and ON are indicated by dotted lines. A) Measurements were taken for WT (BY4724), *imd2Δ::HTB1-EGFP* (FUY2417), and *imd2Δ::HTB1-EGFP sir3Δ* (FUY2428) strains in the logarithmic phase. B) Yeast strains (FUY2417) to which MPA (0.06, 0.09, and 0.15 µg/mL) was added were sampled and analyzed over time (1, 2, and 4 h). Samples without MPA are shown in gray and those with MPA are shown in blue with the data overlaid.

*IMD2* is epigenetically regulated in a GTP-dependent manner, and its expression varies between cells. Our previous single-cell tracking analysis revealed that ∼30–40% of cells exhibited expression even before GTP depletion (13). Flow cytometry (FCM) was used to analyze the heterogeneous expression of *IMD2* in the cell populations. To analyze *IMD2* expression at the single-cell level using enhanced green fluorescent protein (EGFP), we constructed a yeast strain (FUY2417) in which the *IMD2* promoter was preserved and its ORF was replaced with that of *HTB1-EGFP*. *IMD2* is repressed by the Sir protein complex, and this Sir-dependent repression is lifted upon deletion of *SIR3* (13). To distinguish between the suppressed (OFF) and expressed (ON) states of *IMD2*, we used the nonfluorescent wild-type (WT) strain BY4742 as the OFF control and the *imd2Δ::HTB1-EGFP sir3Δ* strain (FUY2428) as the ON control. The threshold was determined as the median fluorescence intensity between the top 5% of BY4742 and the bottom 5% of FUY2428. The analysis revealed that a part of the cell population in the yeast strain (FUY2417) exhibited fluorescence above the threshold for OFF expression, indicating that *IMD2* is expressed in some cells even prior to GTP depletion, as the expression distribution consisted of both OFF and ON cells (Fig. 1A). As *IMD2* expression is induced by MPA, an inhibitor of de novo GTP biosynthesis, we added varying concentrations of MPA (0.06, 0.09, and 0.15 μg/mL) and analyzed the concentration-dependent changes in *IMD2* expression at 1, 2, and 4 h after treatment. The results showed that 1 h after MPA addition, a bimodal distribution with distinct ON and OFF peaks was observed at all concentrations (Fig. 1A). This suggests that even within a single population, there is a mixture of cells that respond rapidly to MPA and those whose response is delayed. From 2 h onward, the overall distribution shifted to the ON state. At 0.06 μg/mL MPA, *IMD2* expression was heterogeneous, with a mixture of ON and OFF cells. This heterogeneity was resolved by increasing the MPA concentration, which led to a higher proportion of ON cells. At 0.15 μg/mL, the majority of the population expressed *IMD2* (Fig. 1B).

### *IMD2* shows different responses to GTP depletion in ON and OFF expression cell populations

**Figure 2:**
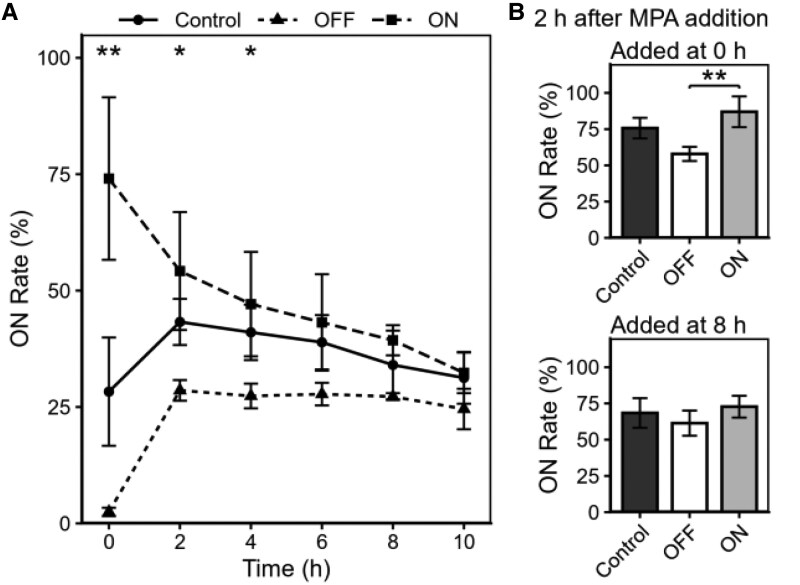
*IMD2* shows different responses to GTP depletion in ON and OFF expression cell populations. A) Yeast strains (FUY2417) in the logarithmic phase were analyzed by FACS, and cells were sorted into expression ON and OFF populations based on the OFF and ON thresholds. As a control, a population was sorted from the entire area, regardless of fluorescence intensity. After sorting, the cells were cultured and analyzed over time using FCM (Fig. S1A). Analysis was performed on 1,000 cells from each sample, and the proportion of cells exhibiting fluorescence intensity above the threshold (ON rate) was calculated and plotted as a line graph. Data are presented as mean ± SD from three independent biological replicates. Statistical significance was evaluated using one-way ANOVA (*P < 0.05, **P < 0.01). B) MPA (0.15 µg/mL) was added to the sorted samples (at 0 and 8 h), and samples were analyzed by FCM after 2 h of incubation. Analysis was performed on 1,000 cells from each sample, and the ON rate was calculated and plotted as a bar graph. Data are presented as mean ± SD from three independent biological replicates. Statistical significance was evaluated using one-way ANOVA, followed by Tukey's multiple comparison test (*P < 0.05, **P < 0.01).

Because the heterogeneity of *IMD2* expression is lost in the *sir3Δ* strain (13), we reasoned that the mechanism of Sir-dependent repression generates this heterogeneity. To test this hypothesis, we investigated whether the heterogeneous expression state of *IMD2* observed during GTP depletion depended on the initial expression state (ON or OFF) before GTP depletion was induced. Using fluorescence-activated cell sorting (FACS), the ON- and OFF-expression cell populations were separated from the total cell population, and FCM analysis was performed (Fig. S1A). After separating the cell population into ON and OFF groups, cultivation was initiated, and FCM analysis was performed over time. As a result, the populations converged to an equilibrium state over time, characterized by the coexistence of ON and OFF cells (Fig. S1B, left panel). The quantification of the ON rates in each sample also showed that over time, both the ON and OFF cell expression groups converged toward the values of the control group (Fig. 2A). In addition, MPA was added to the ON and OFF cell groups at 0 and 8 h after separation, and analysis was performed 2 h after cultivation. As a result, a difference in expression induction between the ON and OFF cell groups was observed 2 h after cultivation, but only in the samples to which MPA had been added immediately (at the 0 h time point) after separation (Fig. S1B, right panel). In the ON cell group, the expression distribution shifted to the right, indicating that the entire cell population was uniformly induced to express. In contrast, in the OFF cell group, both induced and noninduced cells were mixed, revealing that in a part of the OFF group, induction of expression was inhibited. These differences in responsiveness were also confirmed to be statistically significant between the two groups in terms of the quantified ON rate (Fig. 2B). In contrast, the significant difference in MPA responsiveness between the two groups disappeared when MPA was added, and the sample was cultured for 2 h after the expression states shifted to equilibrium (8 h after separation). These findings clarify that *IMD2* is regulated in a cell expression state-dependent manner during GTP depletion.

### *IMD2* is regulated in cell populations depending on its expression state just before GTP depletion

**Figure 3:**
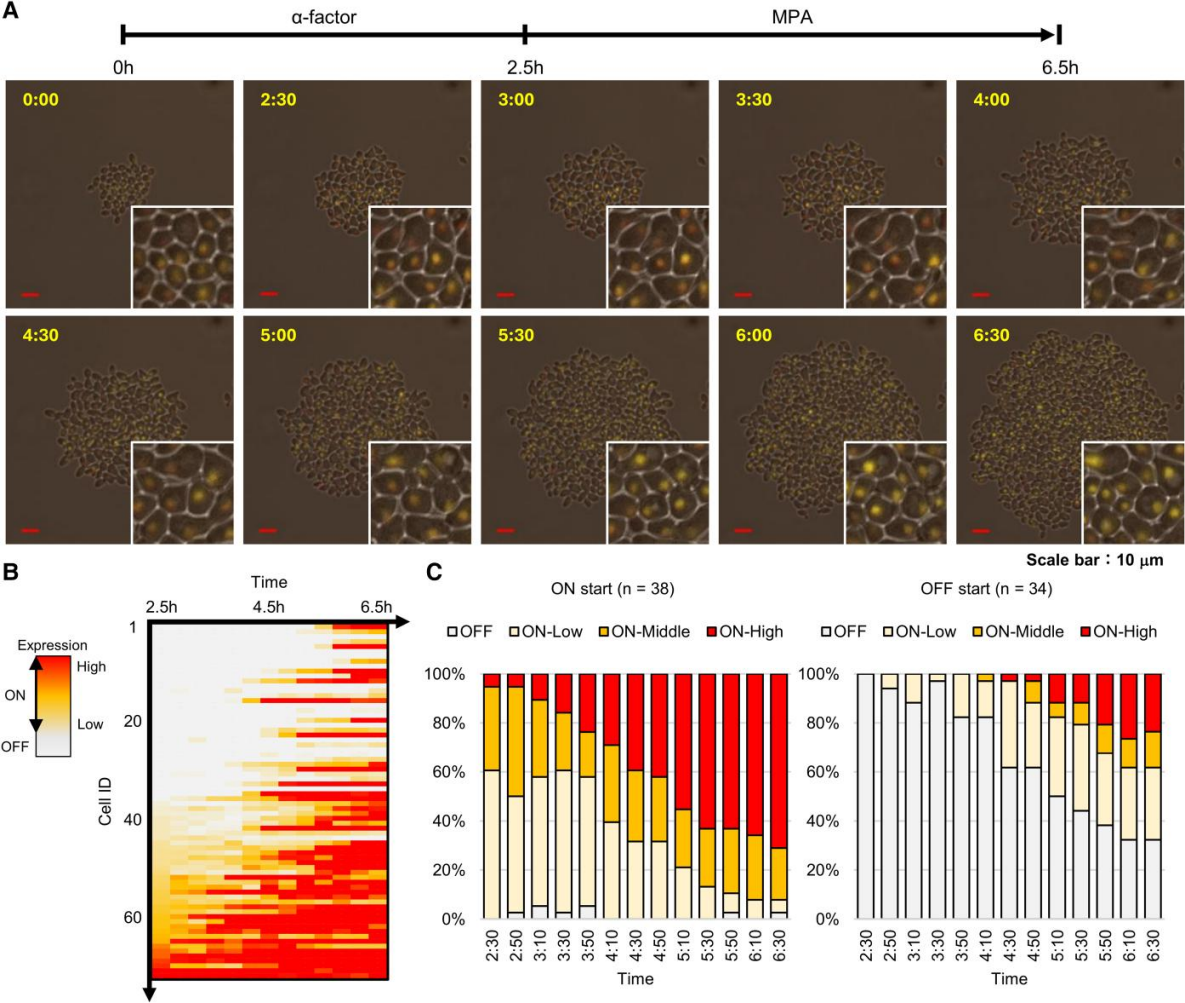
*IMD2* is regulated in cell populations depending on its expression state just before GTP depletion. A) Image tracking of a single cell of the yeast strain (FUY1961). Images were captured every 20 min over a period of 6.5 h, and EYFP images were overlaid with bright-field images. For the first 2.5 h, alpha factor was added at 0.05 ng/mL, and for the remaining 4 h, MPA was added at 0.10 µg/mL. Scale bar: 10 µm. The photograph in the lower right corner of each image is a magnified view of the tracking of some of the cells. B) The expression states of 72 yeast cells (FUY1961), which were synchronized with alpha factor and then released from synchronization with the addition of MPA, were tracked over 4 h for each individual cell. The vertical axis represents each cell, and the horizontal axis represents the time course of tracking. The expression states are indicated by color changes: OFF (gray), ON-Low (light orange), and ON-High (red), indicating intensity. The intensity scale was capped at 2.0, with all values ≥2.0 displayed in red. C) For the 72 cells analyzed in (B), the expression states at the time of MPA addition were classified into two categories (*IMD2* expression <0.5: OFF start, 0.5 or more: ON start). Changes in the proportion of expression states within each cell group. *IMD2* expression levels were divided into four categories (*IMD2* expression <0.5: OFF [gray], 0.5 to <1.25: ON-Low [light orange], 1.25 to <2.0: ON-Middle [orange], ≥2.0: ON-High [red]), and the proportion of each expression state is indicated with a stacked bar graph. The left panel shows the stacked bar graph for the 38 cells that were ON at the time of MPA addition, and the right panel shows the stacked bar graph for the 34 cells that were OFF at the time of MPA addition.

Next, we investigated how the expression state of *IMD2* and the cell cycle phase immediately before GTP depletion affected the induction of *IMD2* expression upon GTP depletion. Synchronization at the G1 phase was performed using alpha factor to align the cell cycles. After synchronization, the cells were released and MPA was added simultaneously to induce GTP depletion in the synchronized cell population. Changes in *IMD2* expression were analyzed at the single-cell level using a time-lapse tracking system (12). For the analysis, we used a *bar1Δ* strain (FUY1961), which is a derivative of the yeast strain (FUY1735) previously employed in our single-cell studies (13). The use of the *bar1Δ* strain prevents the proteolytic degradation of alpha factor by the Bar1 protease, thereby maintaining higher steady-state concentrations of the peptide in the medium. After culturing the FUY1961 strain for 6 h, alpha factor (0.05 ng/mL) was added, and the culture was continued for 2.5 h to synchronize cells in the G1 phase. Next, the medium was replaced with one containing 0.10 μg/mL MPA, thereby releasing the synchronization and inducing expression at the same time (Fig. 3A; Movie S1). Cells present at the time of alpha-factor addition were tracked at 20 min intervals for 4 h, and the expression levels of all 72 cells present at the time of MPA addition were quantified (Fig. 3B). The analysis revealed that while an increase in expression levels was confirmed in most cells, some cells did not show any increase. Interestingly, the difference in whether expression was induced could not be explained solely by the initial expression levels immediately before MPA addition. To evaluate the heterogeneity of the expression induction, we calculated the percentages of ON and OFF cells. To track the responsiveness in the ON cell group, we further subdivided the expression levels into stages (low, middle, and high). The highest threshold for ON-High was set based on the maximum expression intensity at the time of MPA addition. This approach allowed us to capture gradual changes in expression in OFF cells without being overly influenced by a few highly expressing cells. Analysis showed that the proportion of expression ON cells in the entire population increased upon MPA addition, and after 4 h of MPA treatment, the proportion of cells with high-expression levels increased (Fig. S2A). To examine how the pre-GTP depletion expression state affects *IMD2* induction by MPA, cells were grouped according to their expression states immediately before MPA addition into an ON cell group of 34 cells (Fig. S2B, left panel) and an OFF cell group of 38 cells (Fig. S2B, right panel), and expression states after MPA addition were tracked. Both the ON and OFF groups showed variability in expression timing within each group, and differences in sensitivity to MPA were observed between the ON and OFF groups. In the ON group, the proportion of “ON-High” cells with high-expression intensity increased by 71.0% after 4 h of MPA addition (Fig. 3C, left panel), whereas in the OFF group, it increased by only 23.5% (Fig. 3C, right panel), and the timing of the increase in highly expressing cells tended to be delayed.

These results demonstrate that, compared with the OFF group, the ON group exhibited greater sensitivity to MPA, indicating that *IMD2* is subject to MPA-dependent regulation. However, even under conditions of synchronized cell cycle and expression state, variability in the time required for induction was observed within each cell group, suggesting that factors other than the expression state of *IMD2* contribute to *IMD2* expression control during GTP depletion.

### In *IMD2*, prior up–down expression experiences before GTP depletion suppress expression induction at the time of GTP depletion

**Figure 4:**
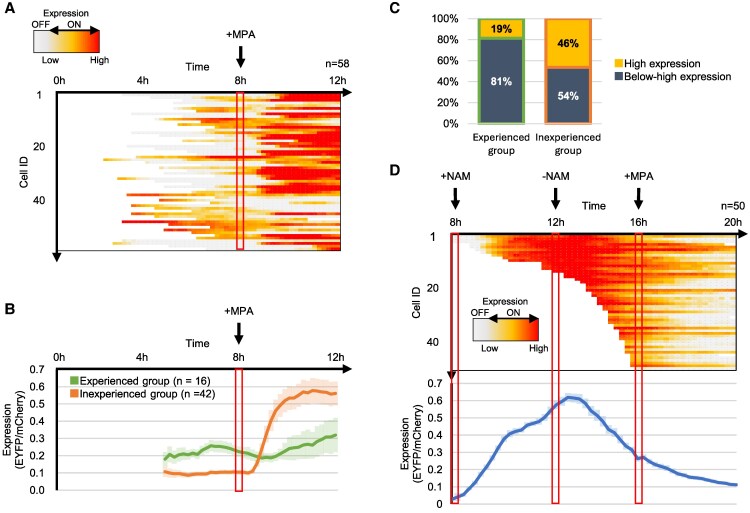
In *IMD2*, prior up–down expression experience before GTP depletion suppressed gene induction upon GTP depletion. A) Yeast strain (FUY1735) was tracked at the single-cell level. Single-cell tracking was performed on four lineages, and the expression states before and after MPA addition were tracked, resulting in the expression state analysis of 58 cells. The vertical axis represents the change in the expression state of each cell, and the horizontal axis shows the tracking time. The expression state intensity is indicated by color changes: OFF (gray), ON-Low (light orange), and ON-High (red). B) Changes in the average expression levels of each cell group are shown as line graphs. The horizontal axis represents time, and the vertical axis represents the expression levels. Error bars indicate SEM. Of the 58 cells in (A), 16 cells that exhibited up–down expression, in which the expression level increases once and the decreases before MPA addition, were classified as the “expression experienced group,” while 42 cells that did not were classified as the “expression inexperienced group.” The experienced and inexperienced groups are represented by green and orange, respectively. C) Expression ratios of each cell group defined in (B) 4 h after MPA addition. The expression state of MPA was classified into two levels (*IMD2* expression <0.5: below high expression [dark blue], ≥0.5: high expression ON [orange]), and the proportion of each state is shown in a stacked bar graph. D) Expression state analysis results. After 8 h of cultivation in YPD, cells were cultivated for 4 h in YPD medium containing 5 mM nicotinamide (NAM). Next, after 4 h in YPD medium, the cells were further cultured in a medium containing 0.06 μg/mL MPA. Upper panel: Changes in the expression of individual cells. The vertical axis represents the change in the expression state of each cell, and the horizontal axis shows the tracking time. The expression state intensity is indicated by color changes: OFF (gray), ON-Low (light orange), and ON-High (red). Lower panel: Changes in the expression levels of individual cells are shown as line graphs. The horizontal axis represents time, and the vertical axis represents the expression levels. Error bars indicate SEM.

Previous analyses have shown that the kinetics and intensity of the response to GTP depletion differ depending on the state of expression immediately before GTP depletion. However, even among cells with the same expression state prior to GTP depletion, there was variability in their responsiveness after MPA addition. To investigate the factors underlying this heterogeneity, we focused on the transition of expression changes prior to GTP depletion rather than on the expression state of each cell. Because it has been shown that *IMD2* expression states switch even in the absence of GTP depletion (13), we examined how prior up–down expression experiences, in which the expression level increases once and then decreases before GTP depletion, affect the responsiveness under GTP-depleted conditions. Yeast strain (FUY1735) was cultured for 8 h, then transferred to media containing 0.06 μg/mL MPA, and further incubated for 4 h. Four lineages of cells were tracked every 15 min for 12 h, quantifying all 592 cells observed (Movies S2–S5). From these, 58 cells whose expression changes could be tracked before and after MPA addition were selected (Figs. S1 and 4A). Among these 58 cells, 16 that exhibited up–down expression before MPA addition were grouped as the “expression experience” group, while the 42 cells without such experience were classified as the “nonexperience” group (Fig. 4B). A comparison of expression states after MPA addition between the two groups revealed that the induction rate of expression after MPA addition was reduced in the experienced group, and the proportion of high-expression cells 4 h after MPA addition decreased (Fig. 4C). These results suggest that up–down expression experience in the absence of GTP depletion acts to suppress subsequent expression induction.

Next, to examine whether the experience of up–down expression suppresses expression induction under GTP depletion, an operation was performed to force cells to undergo up–down expression before adding MPA. In this experiment, to prevent intercellular heterogeneity arising from endogenous GTP levels, the histone deacetylase inhibitor nicotinamide (NAM), which inhibits Sir2, was used to uniformly induce expression in the cell population. After culturing the yeast strain (FUY1735) for 8 h, cells were incubated for 4 h in media containing 5 mM NAM, then returned to regular media for 4 h, thus forcing *IMD2* expression in the entire population to undergo up–down. Subsequently, cells were cultured for 4 h in media containing 0.06 μg/mL MPA. Cultures were initiated from a single cell, and all cells were tracked every 15 min for 20 h (Movie S6). Expression levels of each cell were quantified, and those present up to 2 h after MPA addition were extracted for analysis (Fig. 4D). The analysis showed that all cells present during NAM treatment went through up–down expression as a result of NAM addition and removal, and suppression of expression induction in response to MPA was observed. Furthermore, there were no cells with high-expression levels 4 h after MPA addition.

Taken together, these results clarify that, in *IMD2*, undergoing up–down expression experiences—resulting from fluctuations in heterochromatin structure—acts to suppress expression induction during GTP depletion.

## Discussion

Previous research on *IMD2* has focused on the regulation of heterochromatin structure and transcription start site control, both of which are dependent on intracellular GTP levels (13, 27, 32). However, the expression of *IMD2* under both GTP-depleted and nondepleted conditions shows heterogeneity among cells, even under the same culture conditions, and the mechanisms by which it changes in response to intra- and extracellular environmental shifts remain unclear. In this study, we demonstrated that epigenetic and transcriptional memory contributes to the regulation of the heterogeneous expression state of *IMD2* in cell populations.

Certain genes possess transcriptional reinduction memory, whereby their responsiveness becomes faster and stronger when subjected to the same stimulus multiple times (33, 34). In budding yeast, transcriptional memory has been studied in highly inducible genes, such as *GAL* genes and *INO1*, and the factors involved have been identified (35–38). Additionally, there is evidence for the memory of a transcriptionally inactive state, allowing cells to suppress the expression of unnecessary genes (39). In this study, we revealed that the *IMD2* gene, which is transcriptionally regulated in response to a decrease in intracellular GTP, acquires a memory system that is distinct from previously known mechanisms. *IMD2* expression fluctuates in response to physiological changes in GTP. In cells where expression level increased and then decreased, a subsequent decrease in GTP suppressed *IMD2* activation. When single-cell tracking was performed for >20 h, cell division became too advanced, causing the cells within the field of view to become densely packed, making continued tracking difficult. Therefore, in this study, it was not possible to track cells until those that had acquired transcriptional memory were once again induced to express, and thus, the duration for which cells maintained transcriptional memory could not be determined. However, by isolating cell populations according to their expression states, it was found that after 8 h of separation, the expression states transitioned to equilibrium, and no differences were observed in responsiveness to MPA (Fig. 2A and B). This suggests that *IMD2* memory is maintained for only ∼8 h. These results can be interpreted as follows: in a subset of expression OFF cell populations, abandoning memory leads to a transition of the expression state to ON, whereas conversely, in a subset of expression ON cell populations, a transition from ON to OFF leads to newly acquired memory. Currently, memory maintenance may depend on both the passage of time and the number of cell divisions. Because the regulation of *IMD2* expression involves deacetylation of histone H4K16 by Sir2 and the actions of histone acetyltransferases (32), it is possible that the epigenetic modification status may be inherited and reorganized through mitosis, reflecting a cell division–related aspect. In addition, dynamic factors, such as the localization of Sir proteins and GTP concentration, change over time, presenting a temporal aspect. Therefore, both may contribute to the maintenance and decay of memory. Additionally, although we have previously reported that the expression state of parent cells does not affect daughter cells (13), the fact that daughter cells arising after NAM removal also showed suppressed expression induction by MPA suggests that memory is transmitted across generations.

The biological significance of suppressing the expression induction accompanying a decrease in intracellular GTP is currently unknown. Although the transcriptional memory observed in *IMD2* appears to contradict environmental adaptation, as excessive GTP can trigger excessive cell division (40), it is possible that the short-term reproduction of GTP is inhibited to maintain cellular homeostasis. Furthermore, in this study, memory formation was induced using NAM to disrupt the heterochromatin structure; therefore, it is also possible that memory is formed independently of the decrease in intracellular GTP.

It has been suggested that *IMD2* is regulated by a three-step mechanism: first-stage epigenetic regulation in response to physiologically fluctuating GTP; second-stage epigenetic regulation in response to reduced GTP due to external stimuli such as MPA; and third-stage sequence-dependent regulation in response to lethal GTP depletion (13). In the first stage of *IMD2* regulation, ∼30–40% of the cells in the population consistently maintain *IMD2* expression in the ON state, and because the overall *IMD2* expression across the entire cell population remains constant, it is possible that transcriptional memory exists not at the single-cell level but to maintain the stability of the collective expression level. This is thought to enhance fitness at the population level in response to GTP depletion. Studies on osmotic stress have shown that even under nonstress conditions, the expression of stress-responsive genes is not uniformly suppressed across the entire cell population; rather, some cells stochastically express these genes (41). It has been revealed that such minority populations of expressing cells demonstrate higher fitness under conditions of osmotic stress.

By tracking *IMD2* expression at the single-cell level, we revealed differences in responsiveness to GTP depletion. In this study, we focused only on cases in which *IMD2* expression showed up–down fluctuations. However, because there are also cells in which expression does not fluctuate, cells in which it only goes up, only goes down, or exhibits down–up patterns, it is necessary to consider the effects of memory under these various expression fluctuation scenarios. However, because the range of expression fluctuation during normal cultivation is very small, it is difficult to detect differences arising from such fluctuations.

Previous reports have suggested that Sir proteins hinder the switching of transcription start sites from upstream to downstream of the RE (13, 27). Indeed, in expression OFF cells, where heterochromatin is formed by Sir proteins, the expression distribution during GTP depletion induced by MPA addition (0.15 μg/mL) was greater than that in expression ON cells, which form euchromatin (Fig. 1). However, single-cell tracking of expression ON cells revealed that although not as pronounced as in the expression OFF cell group, the expression states became uneven (Fig. 2D and E). This suggests that factors other than the expression state control mediated by Sir proteins should be considered. A study using guanine, which is required for the GTP salvage pathway, reported two types of regulation depending on cell density during *IMD2* induction: in the initial phase, cells were insensitive to guanine, but at high cell densities, they became guanine sensitive, regardless of the presence of MPA (42). Additionally, regarding the progression to the expression ON state, possibilities include cells switching from expression OFF to ON, cells maintaining the ON state, and cells just before transitioning from ON to OFF state. Considering the estimated GTP levels in vivo, the first two cases likely have low GTP levels and need to maintain the ON state; thus, within the expression ON cell group, sequence-dependent regulation during GTP depletion may also become uneven. Applying similar reasoning to the OFF cell group, cells that demonstrate fluctuating expression or “up–down” behavior probably have sufficient GTP production, and as a result, transition to the OFF state. However, the range of expression up–down fluctuations without mimicking expression experience is smaller than that observed when mimicking, so it is possible that GTP depletion induced by MPA cannot be fully compensated by this method. Therefore, it is necessary to quantify and compare the actual in vivo GTP levels and *IMD2* expression states. Furthermore, as histone H3K4 methylation and the incorporation of histone H2A.Z are involved in transcriptional memory (43, 44), future research should focus on the state of histones that form chromatin.

## Materials and methods

### Yeast strains and plasmids

All yeast strains used in this study were based on the BY4742 background. A list of the yeast strains used in the experiments is presented in Table 1. The *sir3Δ* strain was generated by replacing the *SIR3* gene with the *KanMX* gene through homologous recombination and was selected on YPD plates containing G418 (200 μg/mL). Fluorescent strains were constructed by digesting pFOM812 (*HTB1-2×mCherry-HIS3*) with *Afe*I, introducing it into the *his3Δ1* locus of BY4742, and selecting on YMD (-histidine) medium. FUY2417 was created by introducing a guide RNA into FUY1735, using CRISPR/Cas9 technology (46) to replace the *HTB1-EYFP* inserted at the *IMD2* locus with *HTB1-EGFP*, and selecting on YPD medium containing Hygromycin (200 μg/mL).

**Table 1:** Saccharomyces cerevisiae strains.

Strain no.	Genotype	Source
BY4742	*MAT@ his3Δ1 lue2Δ0 lys2Δ0 ura3Δ0*	Brachmann et al. (45)
FUY1735	*MAT@ lue2Δ0 lys2Δ0 ura3Δ0 his3Δ1::HTB1-2xmCherry-HIS3 imd2Δ::HTB1-EYFP*	Takahito et al. (13)
FUY1961	*MATa lue2Δ0 lys2Δ0 ura3Δ0 his3Δ1::HTB1-2xmCherry-HIS3 imd2Δ::HTB1-EYFP bar1Δ::KanMX*	This study
FUY2417	*MAT@ lue2Δ0 lys2Δ0 ura3Δ0 his3Δ1::HTB1-2xmCherry-HIS3 imd2Δ::HTB1-EGFP*	This study
FUY2428	*MAT@ lue2Δ0 lys2Δ0 ura3Δ0 his3Δ1::HTB1-2xmCherry-HIS3 imd2Δ::HTB1-EGFP sir3Δ::KanMX*	This study

### Flow cytometry

Yeast strains were cultured in YPD medium at 30 °C until the logarithmic phase (OD(600 nm) = 0.2–1.0) and then collected. The harvested cells were centrifuged, the supernatant was removed, and the cells were resuspended in 1× phosphate-buffered saline (PBS). FCM was performed using BD FACS Melody (BD Biosciences, San Jose, CA, United States). GFP was detected using a 488-nm excitation laser and 527/32 nm emission filter. At least 20,000 cells were analyzed per group. For samples that underwent cell sorting, at least 1,000 cells were analyzed per sample. R was used for the data analysis. The same gating was applied to all cells in the experiment. The OFF threshold was calculated using the following formula from the median fluorescence intensity of the top 5% of BY4742 cells and the bottom 5% of FUY2428 cells.


$$\mathrm{OFF}\;\mathrm{threshold}=\frac{95\;\mathrm{percentile}\;\mathrm{FITC}\text{-}A^{\mathrm{BY}4742}+5\;\mathrm{percentile}\;\mathrm{FITC}\text{-}A^{\mathrm{FUY}2428}}{2}$$

### Cell sorting

Before cell sorting, yeast strains were cultured in YPD medium at 30 °C until the logarithmic phase (OD(600 nm) = 0.2–0.4) and then collected. The harvested cells were centrifuged, and after removing the supernatant, they were resuspended in 1×PBS. For cell sorting, a fluorescence gate was set using WT (BY4742), which was cultured in parallel, and *imd2Δ::HTB1-EGFP sir3Δ* (FUY2428), and an OFF threshold was established. The target yeast strain (*imd2Δ::HTB1-EGFP* (FUY2417)) was sorted in batches of 100,000 cells. As a control, the sample was sorted without setting a fluorescence gate. After sorting, a portion of the sample was analyzed using FCM, and the remainder was centrifuged, the supernatant was removed, and the cells were resuspended in YPD. Subsequently, the culture was incubated at 30 °C and analyzed by FCM.

### Single-cell tracking analysis

The experiments were conducted using previously described methods (12, 13, 47). Yeast strains were cultured in YPD medium at 30 °C to the logarithmic phase (OD(600 nm) = 0.2–1.0) and then harvested. The collected cells were trapped on CellASIC ONIX plates for haploid yeast, and single-cell imaging was performed using a CellASIC ONIX2 Microfluidic System. The ONIX plates were supplied with the medium at a flow rate of 2.0 psi. Single-cell imaging was conducted using an Axio Observer Z1 microscope equipped with a 40× Plan-Neofluar objective lens with a numerical aperture of 1.3. After imaging, the images were analyzed using Axio Vision 4.7.1 (Carl Zeiss) or ZEN 2.3 (blue edition; Carl Zeiss). The expression of *IMD2* (EYFP) was determined using the maximum fluorescence intensity in both the cellular and cell-free (BG: background) regions of each image according to the following formula:


$$IMD2\;\mathrm{expression}=\frac{\mathrm{EYF}P^{\mathrm{in}\;\mathrm{cell}\;\max}-\mathrm{EYF}P^{\mathrm{BG}\;\max}}{\mathrm{mCherr}y^{\mathrm{in}\;\mathrm{cell}\;\max}-\mathrm{mCherr}y^{\mathrm{BG}\;\max}}$$

Alpha factor (0.05 ng/mL), MPA (0.06 or 0.10 μg/mL), and NAM (5 mM) were optionally added.

## Supplementary Material

pgag179_Supplementary_Data

## Date availability

The data that support the findings of this study are available in this manuscript and the Supplementary material.

## References

[1] Grewal SIS . 2023. The molecular basis of heterochromatin assembly and epigenetic inheritance. Mol Cell. 83:1767–1785. 37207657 10.1016/j.molcel.2023.04.020 PMC10309086

[2] Audergon PN, et al 2015. Epigenetics. Restricted epigenetic inheritance of H3K9 methylation. Science. 348:132–135. 25838386 10.1126/science.1260638 PMC4397586

[3] Gartenberg MR, Smith JS. 2016. The nuts and bolts of transcriptionally silent chromatin in *Saccharomyces cerevisiae*. Genetics. 203:1563–1599. 27516616 10.1534/genetics.112.145243 PMC4981263

[4] Rusche LN, Kirchmaier AL, Rine J. 2003. The establishment, inheritance, and function of silenced chromatin in *Saccharomyces cerevisiae*. Annu Rev Biochem. 72:481–516. 12676793 10.1146/annurev.biochem.72.121801.161547

[5] Sun JQ, Hatanaka A, Oki M. 2011. Boundaries of transcriptionally silent chromatin in *Saccharomyces cerevisiae*. Genes Genet Syst. 86:73–81. 21670546 10.1266/ggs.86.73

[6] Thurtle DM, Rine J. 2014. The molecular topography of silenced chromatin in *Saccharomyces cerevisiae*. Genes Dev. 28:245–258. 24493645 10.1101/gad.230532.113 PMC3923967

[7] Oki M, Valenzuela L, Chiba T, Ito T, Kamakaka RT. 2004. Barrier proteins remodel and modify chromatin to restrict silenced domains. Mol Cell Biol. 24:1956–1967. 14966276 10.1128/MCB.24.5.1956-1967.2004 PMC350565

[8] Oki M, Kamakaka RT. 2002. Blockers and barriers to transcription: competing activities? Curr Opin Cell Biol. 14:299–304. 12067651 10.1016/s0955-0674(02)00327-7

[9] Kamata K, Ayano T, Oki M. 2023. Spt3 and Spt8 are involved in the formation of a silencing boundary by interacting with TATA-binding protein. Biomolecules. 13:619. 37189367 10.3390/biom13040619 PMC10136053

[10] Oki M, Kamakaka RT. 2005. Barrier function at HMR. Mol Cell. 19:707–716. 16137626 10.1016/j.molcel.2005.07.022

[11] Mitsumori R, et al 2016. Analysis of novel Sir3 binding regions in Saccharomyces cerevisiae. J Biochem. 160:11–17. 26957548 10.1093/jb/mvw021

[12] Mano Y, Kobayashi TJ, Nakayama J, Uchida H, Oki M. 2013. Single cell visualization of yeast gene expression shows correlation of epigenetic switching between multiple heterochromatic regions through multiple generations. PLoS Biol. 11:e1001601. 23843746 10.1371/journal.pbio.1001601 PMC3699475

[13] Ayano T, Yokosawa T, Oki M. 2024. GTP-dependent regulation of heterochromatin fluctuations at subtelomeric regions in Saccharomyces cerevisiae. Genes Cells. 29:217–230. 38229233 10.1111/gtc.13094 PMC11447825

[14] Hedstrom L . 2009. IMP dehydrogenase: structure, mechanism, and inhibition. Chem Rev. 109:2903–2928. 19480389 10.1021/cr900021w PMC2737513

[15] Hyle JW, Shaw RJ, Reines D. 2003. Functional distinctions between IMP dehydrogenase genes in providing mycophenolate resistance and guanine prototrophy to yeast. J Biol Chem. 278:28470–28478. 12746440 10.1074/jbc.M303736200 PMC3367515

[16] Harrison P, et al 2002. A small reservoir of disabled ORFs in the yeast genome and its implications for the dynamics of proteome evolution. J Mol Biol. 316:409–419. 11866506 10.1006/jmbi.2001.5343

[17] Jenks MH, Reines D. 2005. Dissection of the molecular basis of mycophenolate resistance in *Saccharomyces cerevisiae*. Yeast. 22:1181–1190. 16278936 10.1002/yea.1300

[18] McPhillips CC, Hyle JW, Reines D. 2004. Detection of the mycophenolate-inhibited form of IMP dehydrogenase in vivo. Proc Natl Acad Sci U S A. 101:12171–12176. 15292516 10.1073/pnas.0403341101 PMC514452

[19] Shaw RJ, Wilson JL, Smith KT, Reines D. 2001. Regulation of an IMP dehydrogenase gene and its overexpression in drug-sensitive transcription elongation mutants of yeast. J Biol Chem. 276:32905–32916. 11441018 10.1074/jbc.M105075200 PMC3371605

[20] Davis CA, Ares M Jr. 2006. Accumulation of unstable promoter-associated transcripts upon loss of the nuclear exosome subunit Rrp6p in *Saccharomyces cerevisiae*. Proc Natl Acad Sci U S A. 103:3262–3267. 16484372 10.1073/pnas.0507783103 PMC1413877

[21] Kopcewicz KA, O'Rourke TW, Reines D. 2007. Metabolic regulation of IMD2 transcription and an unusual DNA element that generates short transcripts. Mol Cell Biol. 27:2821–2829. 17296737 10.1128/MCB.02159-06 PMC1899919

[22] Steinmetz EJ, et al 2006. Genome-wide distribution of yeast RNA polymerase II and its control by Sen1 helicase. Mol Cell. 24:735–746. 17157256 10.1016/j.molcel.2006.10.023

[23] Connell Z, Parnell TJ, McCullough LL, Hill CP, Formosa T. 2022. The interaction between the Spt6-tSH2 domain and Rpb1 affects multiple functions of RNA polymerase II. Nucleic Acids Res. 50:784–802. 34967414 10.1093/nar/gkab1262 PMC8789061

[24] Escobar-Henriques M, Daignan-Fornier B, Collart MA. 2003. The critical cis-acting element required for IMD2 feedback regulation by GDP is a TATA box located 202 nucleotides upstream of the transcription start site. Mol Cell Biol. 23:6267–6278. 12917347 10.1128/MCB.23.17.6267-6278.2003 PMC180940

[25] Escobar-Henriques M, Daignan-Fornier B. 2001. Transcriptional regulation of the yeast gmp synthesis pathway by its end products. J Biol Chem. 276:1523–1530. 11035032 10.1074/jbc.M007926200

[26] Jenks MH, O'Rourke TW, Reines D. 2008. Properties of an intergenic terminator and start site switch that regulate IMD2 transcription in yeast. Mol Cell Biol. 28:3883–3893. 18426909 10.1128/MCB.00380-08 PMC2423123

[27] Kuehner JN, Brow DA. 2008. Regulation of a eukaryotic gene by GTP-dependent start site selection and transcription attenuation. Mol Cell. 31:201–211. 18657503 10.1016/j.molcel.2008.05.018

[28] Shaw RJ, Reines D. 2000. *Saccharomyces cerevisiae* transcription elongation mutants are defective in PUR5 induction in response to nucleotide depletion. Mol Cell Biol. 20:7427–7437. 11003640 10.1128/mcb.20.20.7427-7437.2000 PMC86296

[29] Ko MS . 2016. Zygotic genome activation revisited: looking through the expression and function of Zscan4. Curr Top Dev Biol. 120:103–124. 27475850 10.1016/bs.ctdb.2016.04.004

[30] Amano T, et al 2013. Zscan4 restores the developmental potency of embryonic stem cells. Nat Commun. 4:1966. 23739662 10.1038/ncomms2966 PMC3682791

[31] Zalzman M, et al 2010. Zscan4 regulates telomere elongation and genomic stability in ES cells. Nature. 464:858–863. 20336070 10.1038/nature08882 PMC2851843

[32] Ayano T, Oki M. 2024. IMD2, which is located near the boundary of heterochromatin regions, is regulated by the use of multiple HAT-related factors. Genes Genet Syst. 99. 10.1266/ggs.23-00284 38382924

[33] Gialitakis M, Arampatzi P, Makatounakis T, Papamatheakis J. 2010. Gamma interferon-dependent transcriptional memory via relocalization of a gene locus to PML nuclear bodies. Mol Cell Biol. 30:2046–2056. 20123968 10.1128/MCB.00906-09 PMC2849471

[34] Ding Y, Fromm M, Avramova Z. 2012. Multiple exposures to drought ‘train’ transcriptional responses in Arabidopsis. Nat Commun. 3:740. 22415831 10.1038/ncomms1732

[35] Brickner DG, et al 2007. H2a.Z-mediated localization of genes at the nuclear periphery confers epigenetic memory of previous transcriptional state. PLoS Biol. 5:e81. 17373856 10.1371/journal.pbio.0050081 PMC1828143

[36] D'Urso A, Brickner JH. 2014. Mechanisms of epigenetic memory. Trends Genet. 30:230–236. 24780085 10.1016/j.tig.2014.04.004 PMC4072033

[37] Bheda P, et al 2020. Single-cell tracing dissects regulation of maintenance and inheritance of transcriptional reinduction memory. Mol Cell. 78:915–925.e917. 32392469 10.1016/j.molcel.2020.04.016

[38] Li B, et al 2023. Differential regulation of mRNA stability modulates transcriptional memory and facilitates environmental adaptation. Nat Commun. 14:910. 36801853 10.1038/s41467-023-36586-x PMC9936472

[39] Lee BB, et al 2018. Rpd3L HDAC links H3K4me3 to transcriptional repression memory. Nucleic Acids Res. 46:8261–8274. 29982589 10.1093/nar/gky573 PMC6144869

[40] Takizawa Y, et al 2023. Specific inhibitory effects of guanosine on breast cancer cell proliferation. Biochem Biophys Res Commun. 673:67–72. 37356147 10.1016/j.bbrc.2023.06.069

[41] Nadal-Ribelles M, et al 2025. Transcriptional heterogeneity shapes stress-adaptive responses in yeast. Nat Commun. 16:2631. 40097446 10.1038/s41467-025-57911-6 PMC11914649

[42] Shand EL, Sweeney K, Sundling KE, McClean MN, Brow DA. 2024. Live-cell analysis of IMPDH protein levels during yeast colony growth provides insights into the regulation of GTP synthesis. mBio. 15:e0102124. 38940616 10.1128/mbio.01021-24 PMC11323793

[43] Sood V, Cajigas I, D'Urso A, Light WH, Brickner JH. 2017. Epigenetic transcriptional memory of GAL genes depends on growth in glucose and the Tup1 transcription factor in *Saccharomyces cerevisiae*. Genetics. 206:1895–1907. 28607146 10.1534/genetics.117.201632 PMC5560796

[44] Zhou BO, Zhou JQ. 2011. Recent transcription-induced histone H3 lysine 4 (H3K4) methylation inhibits gene reactivation. J Biol Chem. 286:34770–34776. 21849496 10.1074/jbc.M111.273128 PMC3186427

[45] Brachmann CB, et al 1998. Designer deletion strains derived from *Saccharomyces cerevisiae* S288C: a useful set of strains and plasmids for PCR-mediated gene disruption and other applications. Yeast. 14:115–132. 9483801 10.1002/(SICI)1097-0061(19980130)14:2<115::AID-YEA204>3.0.CO;2-2

[46] Cong L, et al 2013. Multiplex genome engineering using CRISPR/Cas systems. Science. 339:819–823. 23287718 10.1126/science.1231143 PMC3795411

[47] Kanada F, Ogino Y, Yoshida T, Oki M. 2020. A novel tracking and analysis system for time-lapse cell imaging of *Saccharomyces cerevisiae*. Genes Genet Syst. 95:75–83. 32249245 10.1266/ggs.19-00061

