# Supplementary material for: In the IMD2 gene of Saccharomyces cerevisiae, the expression memory suppresses the induction of expression during guanosine triphosphate depletion
Source: PNAS Nexus. 2026 May 22;5(5):pgag179. doi: 10.1093/pnasnexus/pgag179 (PMC13220337; doi:10.1093/pnasnexus/pgag179)
Supplement: pgag179_Supplementary_Data [file pgag179_supplementary_data.zip › PNASNEXUS-PNASNEXUS-2026-00169R-s01.docx]

**
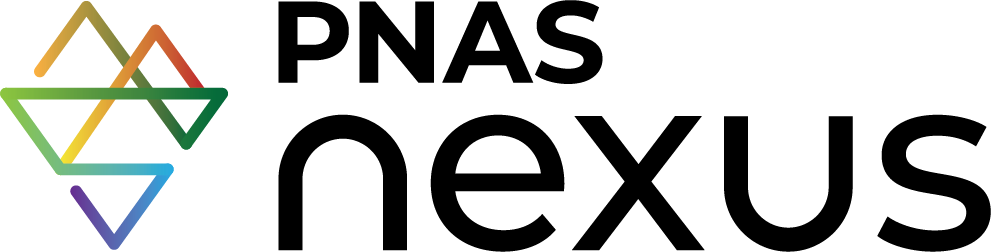
**

**Supplementary Information for**

In the *IMD2* gene of *Saccharomyces cerevisiae,* the expression memory suppresses the induction of expression during GTP depletion.

Takuma Yokosawa, Takahito Ayano, and Masaya Oki

Masaya Oki

Email: ma4sa6ya@u-fukui.ac.jp

**This PDF file includes:**

Supplementary text

Figures S1 to S3

Legends for Movies S1 to S6

SI References

**Other supplementary materials for this manuscript include the following:**

Movies S1 to S6

**
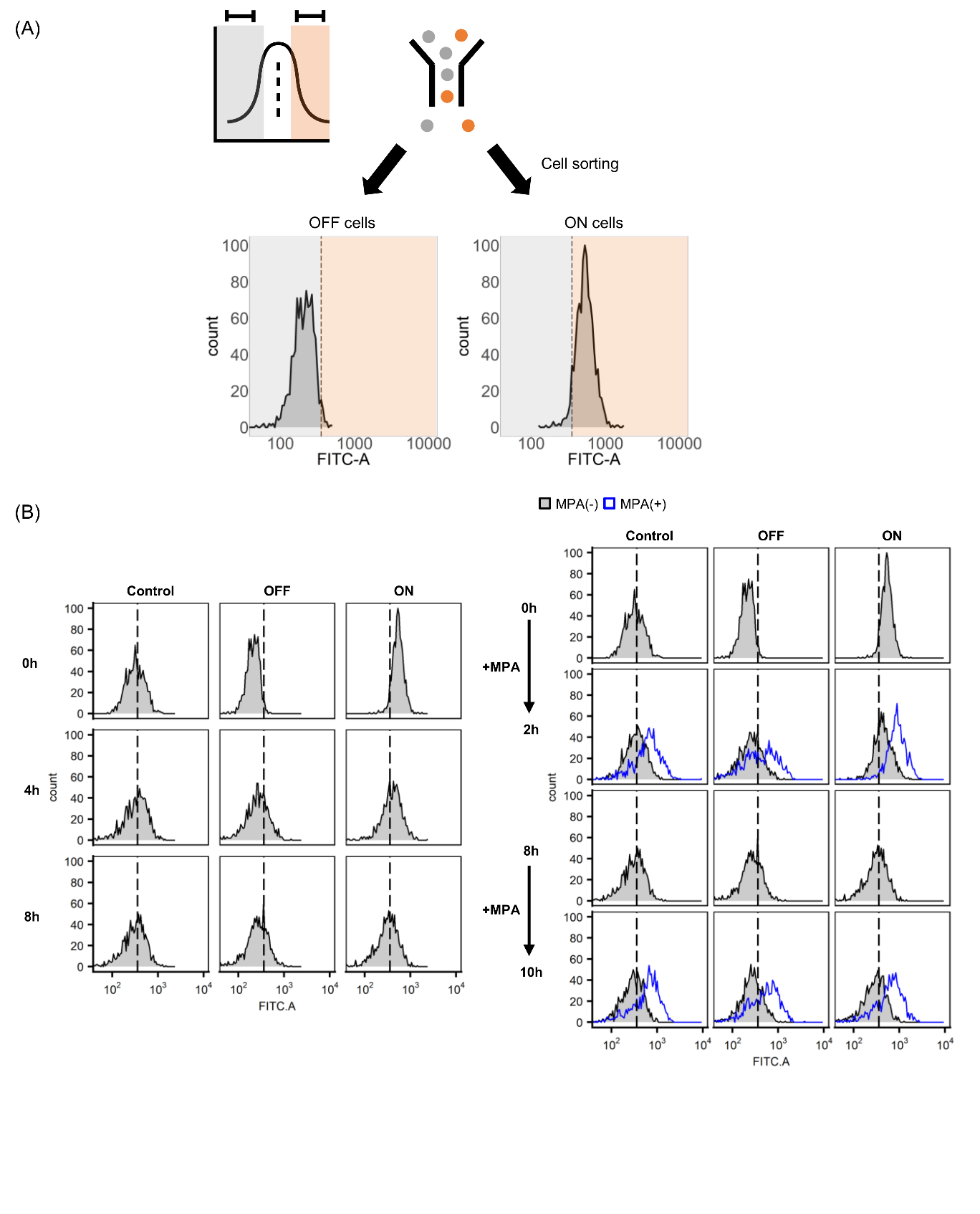
**

**Figure S1. FCM analysis focusing on differences in *IMD2* expression states.** (A) Yeast strains (FUY2417) in the logarithmic phase were analyzed by fluorescence-activated cell sorting (FACS), and cells were sorted into expression ON and OFF populations based on the OFF and ON thresholds. The cells were reanalyzed using flow cytometry (FCM) immediately after sorting. Each sample was analyzed using 1,000 cells and plotted as a histogram. The horizontal axis indicates the fluorescence intensity, and the vertical axis shows the total number of cells. The OFF and ON thresholds are indicated by the dotted lines. (B) The sorted samples from (A) were cultured and analyzed using FCM. Each sample was analyzed with 1,000 cells and plotted as a histogram as in (A). Left panel: The sorted samples from (A) were cultured, sampled over time (0, 4, and 8 h), and analyzed by FCM. Right panel: Samples without MPA addition are shown in gray, and samples with MPA addition are shown in blue, overlaid. MPA (0.15 μg/ml) was added to the sorted samples (at 0 and 8 h), and samples were analyzed by FCM after 2 h of incubation. The figure shows the representative results of three independent experiments. Similar trends were observed in all the experiments.


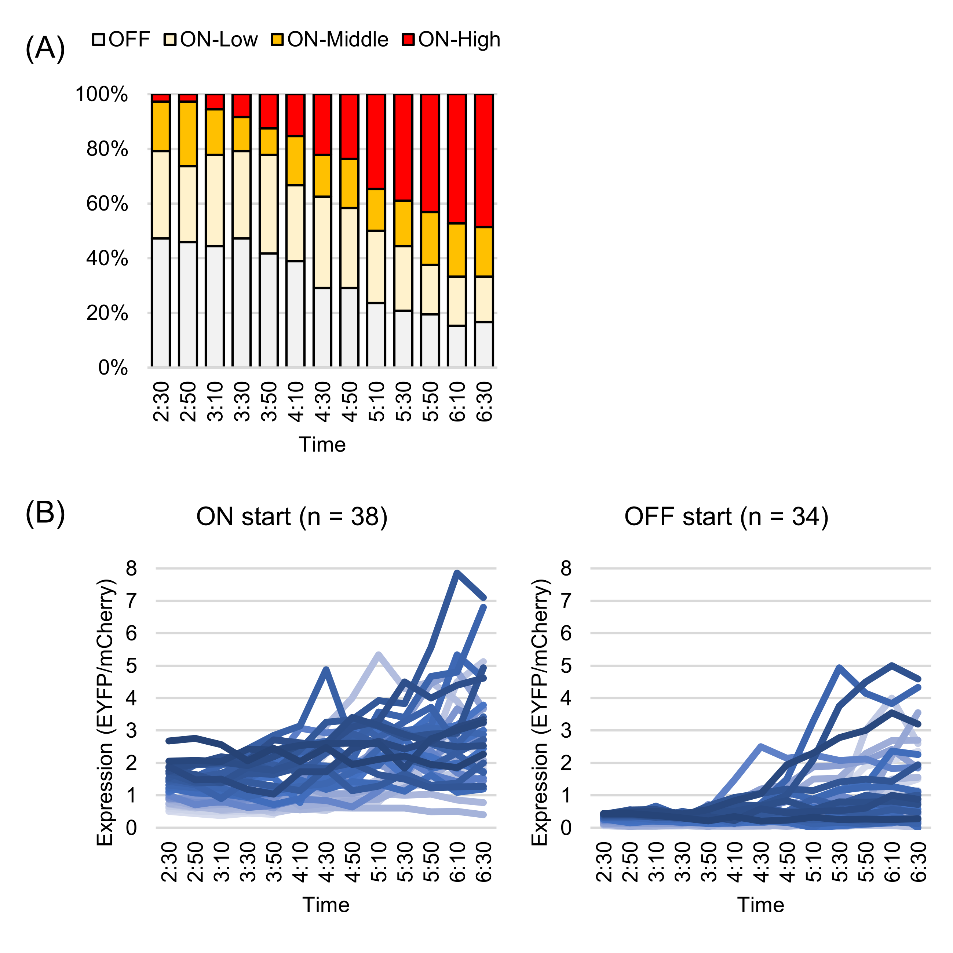


**Figure S2. Single-cell tracking analysis of *IMD2* expression upon MPA treatment after release from cell cycle synchronization.** (A) Changes in the proportion of expression states of 72 yeast cells (strain FUY1961), which were synchronized with alpha-factor and then released from synchronization with the addition of MPA, were tracked over 4 h for each individual cell. *IMD2* expression levels were classified into four categories (*IMD2* expression less than 0.5: OFF (gray), 0.5 to less than 1.25: ON-Low (light orange), 1.25 to less than 2.0: ON-Middle (orange), 2.0 or more: ON-High (red)), and the proportion of each expression state is represented as a stacked bar graph. (B) For the 72 cells analyzed in (A), the expression states at the time of MPA addition were classified into two categories (*IMD2* expression less than 0.5: OFF start, 0.5 or more: ON start). The change in expression states over the 4 h following MPA addition is shown as a line graph. The left panel shows line graphs for the 38 cells whose expression state was ON at the time of MPA addition. The right panel shows line graphs for the 34 cells whose expression state was OFF at the time of MPA addition. The horizontal axis represents time, and the vertical axis indicates the expression level; the line graphs show the expression changes for each cell.


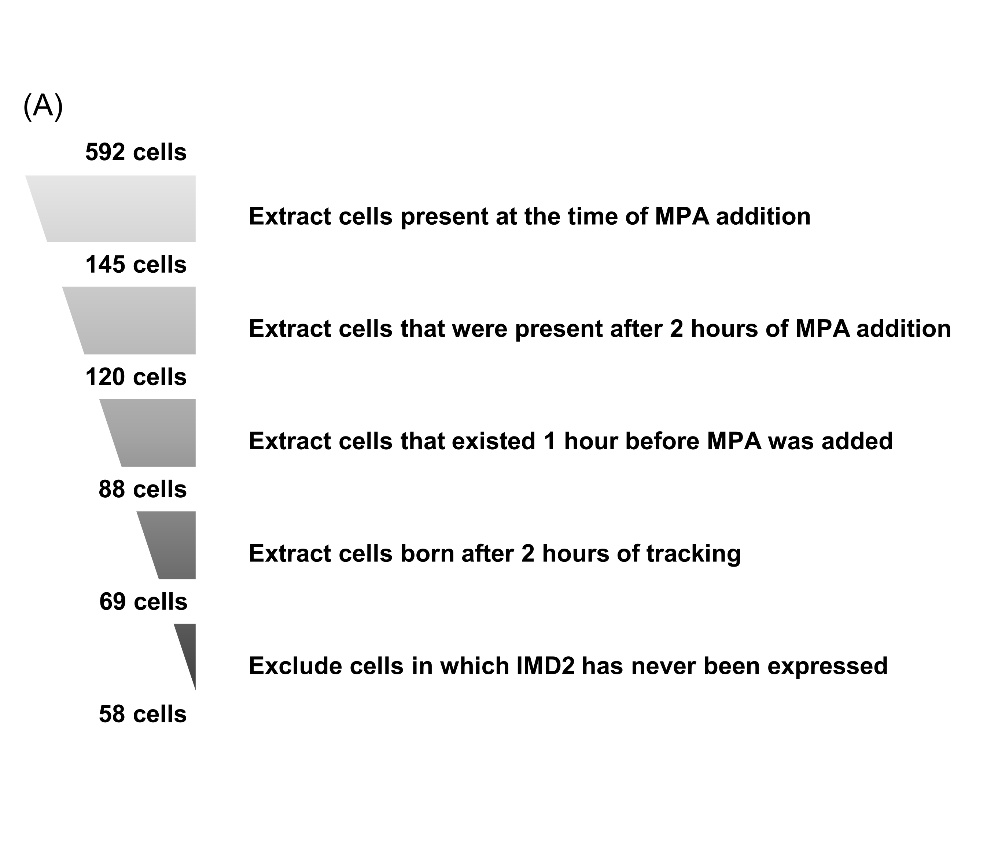


**Figure S3. Flowchart of refining expression experienced and inexperienced groups.** A total of 592 cells were tracked during single-cell tracking, and 145 cells were present at the time of MPA addition. 120 cells were the number present an hour before MPA addition, and 88 cells were the number of cells born two hours after tracking began. After excluding those that never expressed *IMD2* after birth, 58 cells were obtained.

Movie S1 (separate file). Single-cell imaging of response to α-factor and MPA. The experiments were conducted using previously described methods (1-3). The yeast strain (FUY1961) was cultured in YPD medium at 30°C until the logarithmic phase (OD(600 nm) = 0.2–1.0) and collected. The cells were trapped on a yeast-specific CellASIC ONIX plate, and the medium was flowed through a CellASIC ONIX2 Microfluidic System at a flow rate of 2.0 psi. First, cells were cultured in YPD medium for 6 h to increase the cell number in the field of view, followed by live-cell imaging. At the start of imaging, the medium was switched to one containing alpha-factor (0.5 ng/ml) and incubated for 2.5 h. Next, the medium was replaced with one containing MPA (0.10 μg/ml) and cultured for 4 h. Single-cell imaging was performed every 20 min, and the cells present at the time of α-factor addition were tracked for 6.5 h. Single-cell imaging was conducted using an Axio Observer Z1 microscope equipped with a 40x Plan-Neofluar objective lens with a numerical aperture of 1.3. After imaging, the images were analyzed using AxioVision 4.7.1 (Carl Zeiss).

Movie S2 (separate file). Single-cell imaging of response to MPA. The yeast strain (FUY1735) was cultured in YPD medium at 30°C until the logarithmic phase (OD(600 nm) = 0.2-1.0) and collected. The cells were trapped on a yeast-specific CellASIC ONIX plate, and the medium was flowed through a CellASIC ONIX2 Microfluidic System at a flow rate of 2.0 psi, followed by live cell imaging. At the start of imaging, the cells were cultured in YPD medium for 8 h, then switched to a medium containing MPA (0.06 μg/ml) and cultured for 4 h. Live cell imaging was performed at 15-minute intervals, and all cells were tracked. Single-cell imaging was conducted using an Axio Observer Z1 microscope equipped with a 40x Plan-Neofluar objective lens with a numerical aperture of 1.3. After imaging, the images were analyzed using ZEN 2.3 (Blue Edition) (Carl Zeiss).

Movie S3 (separate file). Biological replication of MovieS2 (n=2).

Movie S4 (separate file). Biological replication of MovieS2 (n=3).

Movie S5 (separate file). Biological replication of MovieS2 (n=4).

Movie S6 (separate file). Single-cell imaging of response to NAM and MPA. The experimental setup and microfluidic conditions were identical to those described in Movie S2, except for the following culture conditions. At the start of imaging, the cells were cultured in YPD medium for 8 h, then switched to a medium containing NAM (5 mM) and cultured for 4 h. The cells were then cultured again in YPD medium for another 4 h, after which the medium was switched to one containing MPA (0.06 μg/ml) and cultured for 4 h.

**SI References**

1. Y. Mano, T. J. Kobayashi, J. Nakayama, H. Uchida, M. Oki, Single cell visualization of yeast gene expression shows correlation of epigenetic switching between multiple heterochromatic regions through multiple generations. *PLoS Biol* **11**, e1001601 (2013).

2. F. Kanada, Y. Ogino, T. Yoshida, M. Oki, A novel tracking and analysis system for time-lapse cell imaging of Saccharomyces cerevisiae. *Genes Genet Syst* **95**, 75-83 (2020).

3. T. Ayano, T. Yokosawa, M. Oki, GTP-dependent regulation of heterochromatin fluctuations at subtelomeric regions in Saccharomyces cerevisiae. *Genes Cells* 10.1111/gtc.13094 (2024).
